# A Structure-Toxicity Study of Aß_42_ Reveals a New Anti-Parallel Aggregation Pathway

**DOI:** 10.1371/journal.pone.0080262

**Published:** 2013-11-11

**Authors:** Hélène Vignaud, Claude Bobo, Ioan Lascu, Karin Margareta Sörgjerd, Tamotsu Zako, Mizuo Maeda, Benedicte Salin, Sophie Lecomte, Christophe Cullin

**Affiliations:** 1 Institut de Biochimie et Génétique Cellulaires, CNRS UMR 5095, Université Bordeaux Segalen, Bordeaux, France; 2 Bioengineering Laboratory RIKEN Institute, Wako, Saitama, Japan; 3 Chimie et Biologie des Membranes et Nano-objets, CNRS UMR 5248, Université Bordeaux 1, IPB, Pessac, France; Universitat Autònoma de Barcelona, Spain

## Abstract

Amyloid beta (Aβ) peptides produced by APP cleavage are central to the pathology of Alzheimer’s disease. Despite widespread interest in this issue, the relationship between the auto-assembly and toxicity of these peptides remains controversial. One intriguing feature stems from their capacity to form anti-parallel ß-sheet oligomeric intermediates that can be converted into a parallel topology to allow the formation of protofibrillar and fibrillar Aβ. Here, we present a novel approach to determining the molecular aspects of Aß assembly that is responsible for its *in vivo* toxicity. We selected Aß mutants with varying intracellular toxicities. *In vitro*, only toxic Aß (including wild-type Aß_42_) formed urea-resistant oligomers. These oligomers were able to assemble into fibrils that are rich in anti-parallel ß-sheet structures. Our results support the existence of a new pathway that depends on the folding capacity of Aß .

## Introduction

Amyloid proteins and peptides form a large group of unrelated proteins. Some of these proteins are used for their biophysical properties (e.g., spidroin, the major spider silk protein [1]) or, their capacity to mimic a loss- of -function phenotype (e.g.,like Ure2p in *S. cerevisiae*, responsible for the [URE3] prion phenotype [2]). Amyloid proteins are also involved in many different cellular functions [3,4]. Furthermore, amyloid assembly may also be a convenient mechanism for storing peptide and protein hormones in the secretory granules of the endocrine system [5]. These amyloid structures are formed by the stacking of parallel or anti-parallel β-strands into β-sheets that are orthogonal to the fibril axis and are often stabilized by the lateral association of several different sheets. Despite these positive roles, amyloids are most often studied in the context of disease, particularly neurodegenerative diseases. This is mainly due to the detrimental roles of the huntingtin, alpha-synuclein, prion, and amyloid beta proteins in Huntington’s, Parkinson’s, Creutzfeldt-Jakob, and Alzheimer’s diseases, respectively. Understanding the toxicity of these amyloids is a challenging goal and is being pursued by many scientists. 

Amyloid proteins assemble *via* structurally and kinetically common mechanisms involving monomers (which are often unstructured for *in vitro* experiments [6]), oligomers (which often share structural characteristics that are recognized by specific antibodies, independent of their primary structure [7]), protofibrils, and fibrils. The amyloid auto-assembly process is a matter of great interest because the toxicity of amyloid proteins is clearly related to their fibrillation properties. The concept that toxic intermediates are responsible for cell death has become dogma in the field [8,9], fueling interest in isolating these intermediates. 

This search has indeed been intensive in the field of Alzheimer’s disease research. The metabolism of APP and, in particular, the rate of production of Aß peptides (39-43 residues) are thought to be key events in the pathological process [10,11]. Studies of Aß aggregation have strengthened the link between amyloid intermediates and disease. The identification of pathogenic point mutations within Aß sequences allowed researchers to focus on these intermediates rather than fibrils [12,13]. Since that time, many different intermediates of varying size (from two to hundreds of Aß molecules) and structure (both parallel and anti-parallel ß-sheets) have been suspected to act as “partners in crime” [14,15]. However, the lack of standardized methods [16] and the difficulty of linking *in vitro* biochemical observations to *in vivo* toxicity make it difficult to reconcile these disparate results. 

In this study, we used a biological system that permits structure-toxicity studies [17]. This approach has been successfully used in our lab for another amyloid peptide [18] and allowed us to highlight the role of anti-parallel ß-sheet amyloids in cellular toxicity [19]. We were thus able to address the following question: what distinguishes a harmless Aß_42_ from a toxic Aß_42_? We performed an *in vivo* screen without any *a priori* information about the biochemical nature of the mutations and isolated several Aß4_2_ single mutants varying from harmless to very damaging. The *in vitro* analysis of these mutants led to the identification of a new pathway based on anti-parallel ß-sheet organization. This pathway causes the formation of fibrils that are structurally distinct from the classical-fibrils with parallel in-register structure of Aß [20].

## Materials and Methods

### Yeast strains, media, and plasmids

The yeast strain used was BY4742 (*MAT a, his3∆1, leu2∆0, lys2∆0, ura3∆0*). Yeast cells were grown in SD medium (0.67 % yeast nitrogen base, 2 % dextrose) or SG medium (0.67 % yeast nitrogen base, 2 % galactose) supplemented with 0.67 % casaminoacids or with 20 mg/L histidine (H), 20 mg/L lysine (K) and 60 mg/L leucine (L). 

All expression vectors used were derived from pYe ßYGFP [17], a multicopy yeast-expression plasmid with the selectable *URA3* marker and a *GAL10* promoter in a pYeHFN2U backbone [21]. A PvuII site was inserted between the pre-pro-sequence of alpha factor and GFP by overlapping PCR using pYeAßGFP and pYeαGFP as templates for the primers 705, 706, 866 and 867 (Table 1). This allowed the amplification of a PGAL-α-PvuII-YGFP fragment, which was cloned into the *Bam*HI-*Eco*RI sites of pYeAßGFP. Expression vectors of 〈Aß and 〈Aßarc without GFP were created by PCR amplification from pYe〈AßYGFP or pYeαAßarcYGFP templates using 705 and 879 primers. Fragments were cloned into the BamHI*-*Bsu36I sites of pYeαAßYGFP. Expression vector of 〈Aß_G37C_ without GFP was created by PCR amplification from pYeαAß_G37C_YGFP (see *Mutagenesis*) using 705 and 960 primers. The PCR product was introduced by gap repair method into a PvuII-linearized pYeα-*PvuII*-YGFP 2U plasmid. The empty vector used as control is pYeHFN2U. For the co-transformation experiments, the selectable marker *URA3* was shuffled into the *LEU2* cassette coming from pFL36 [22]

**Table 1:** List of primers.

number	Sequence	
705	5'-GGATGGCCAGGCAACTTTAG-3'	
706	5'-TTTACACTTTATGCTTCCGG-3'	
861	5'-GTGACCATTAACATCACC-3'	
866	5'-GAGGCTGAAGCTGCAGCTGGTATGTCTAAAGGTGAAGAATTATTC-3'	
867	5'-CTTCACCTTTAGACATACCAGCTGCAGCTTCAGCCTCTCTTTTATC-3'	
879	GCGAATTCCTTAGGTTACGCTATGACAACACCGC-3’	
902	5'-TATTGCCAGCATTGCTGC-3'	
960	5’AGTAGAGACATGGGAGATCCCCCGCGAATTCCTTAGGTTACGCTATGACAACACCG-3	
980	5'-CCGACATGACTCAGGATATGAAnnnCATCATCAAAAATTGGTG-3'	
981	5'-CACCAATTTTTGATGATGnnnTTCATATCCTGAGTCATGTCGG-3'	
984	5'-GTGGGTTCAAACAAAGGTGCAnnnATTGGACTCATGGTGGG-3'	
985	5'-CCCACCATGAGTCCAATnnnTGCACCTTTGTTTGAACCCAC-3'	
986	5'-CAAAGGTGCAATCATTGGAnnnATGGTGGGCGGTGTTGTCATAG-3'	
987	5'-CTATGACAACACCGCCCACCATnnnTCCAATGATTGCACCTTTG-3'	
939	5’-CATCGGTCTGATGGTTTGCGGCGTTGTGATCGCTTAATAGG-3’
940	5’CCTATTAAGCGATCACAACGCCGCAAACCATCAGACCGATG-3’
978	5’GAAGCTGGTGTTCTTGCTGGTGACGTGGGTTCTAACAAGGG-3’
979	5’CCCTTGTTAGAACCCACGTCACCAGCAAGAACACCAGCTTC-3’
1008	5’-CGATCACAACGCCACCAACCATGGTACCGATGATAGCACC-3’
1009	5’-GGTGCTATCATCGGTACCATGGTTGGTGGCGTTGTGATCG-3’

The expression vector for Aß_42_ in *E. coli* was a kind gift from D. Walsh and was described previously as Aß (M1-42) [23]. Substitutions were introduced by site-directed mutagenesis using the QuikChange® Site-Directed Mutagenesis Kit (Stratagene) according to the supplier’s recommendations. The primers used are shown in Table 1.

### Mutagenesis

The Aß sequence was amplified by PCR from pYeαAßYGFP using primers 861 and 902 (Table 1). A Taq DNA polymerase with no proofreading activity (New England Biolabs M0237 Taq DNA polymerase) was used under error-prone reaction conditions (50 mM KCl, 10 mM Tris-HCl pH8.3, 4.76 mM MgSO_4_, 0.5 mM MnCl_2_) with the following nucleotide concentrations: 0.09 mM dCTP, 0.06 mM dATP, 0.14 mM dTTP, and 0.02 mM dGTP. The corresponding PCR products were cloned by the gap repair method into a PvuII-linearized pYeα-PvuII-YGFP2U plasmid.

The mutagenesis of “non-toxic” residues was performed using the QuikChange® Site-Directed Mutagenesis Kit according to the supplier’s recommendations. The degenerate primers used are shown in Table 1 (primers 980 and 981 for valine 12 mutagenesis, 984 and 985 for isoleucine 31, and 986 and 987 for leucine 34). 

### Isolation of toxic mutants

The library obtained after the gap repair was plated onto SD casa medium. After replication on SG HKL medium, colonies that exhibited growth defects were isolated and spotted individually. In this study, 6,000 clones were analyzed, and 39 were confirmed to have a toxic phenotype. The plasmids were then extracted and re-transformed into *S. cerevisiae* after an amplification step in *E.coli*. Clones with interesting phenotypes were sequenced. 

### Spotting assay

Tenfold serial dilutions starting with equal numbers of cells (10^6^ cells) were performed in sterile water. The cells for spotting assays were derived from a pool of ten independent fresh transformants; 10 µL drops were loaded on the appropriate SD and SG media.

### Protein extraction and western blotting

Alkaline lysis was used for protein extraction. Five OD units of yeast cells in the exponential growth phase were permeabilized with 500 µL of 0.185 M NaOH and 0.2 % ß-mercaptoethanol. After 10 min on ice, trichloroacetic acid (TCA) was added to a final concentration of 5 %. Samples were incubated for an additional 10 min on ice. Precipitates were collected by centrifugation at 13,000 g for 5 min, and pellets were dissolved in 35 µL of dissociation buffer (4 % SDS, 0.1 M Tris-HCl pH 6.8, 4 mM EDTA, 20 % glycerol, 2 % ß-mercaptoethanol and 0.02 % bromophenol blue) and 15 µL of 1 M Tris-base. 

Extracts were incubated for 5 min at 100 °C and separated by SDS-PAGE in a 12 % polyacrylamide gel. Gels were either strained with PageBlue Protein Straining Solution (Fermentas) according to supplier’s recommendations, or transferred on nitrocellulose membrane. After semi-dry transfer, the membranes were probed with monoclonal anti-Aß antibodies (Santa-Cruz Biotechnology). Peroxidase-conjugated anti-mouse antibodies (Sigma) secondary antibodies were used. Binding was detected with the SuperSignal reagent and the G:Box system (Syngene).

### Fluorescence microscopy

Cells in the exponential growth phase were washed in water and resuspended in SG medium. After 8 h, cells were observed with an Axioskop 2 plus fluorescence microscope (Zeiss) coupled with an AxioCam black and white camera (Zeiss). The LP-GFP filter was used.

### Aß expression and purification

A ZYM-5052 auto-inductive system was used for Aß expression [24]. Briefly, several clones of transformed BL21 DE3 PLys S were grown on 10 mL LB medium preculture that contained 1 % dextrose, 100 mg/L ampicillin, and 25 mg/L chloramphenicol. A one-day preculture was added to 990 mL of ZYM 5052 medium (1 % N-Z-amine, 0.5 % yeast extract, 25 mM Na_2_HPO_4_, 25 mM KH_2_PO_4_, 50 mM NH_4_Cl, 5 mM Na_2_SO_4_, 2 mM MgSO_4_, 0.5 % glycerol, 0.05 % dextrose, 0.2 % lactose) containing 100 mg/L ampicillin and 25 mg/L chloramphenicol and incubated overnight at 37 °C.

Cell pellets were resuspended at 1 g per 2.5 mL of TE buffer (50 mM Tris, 1 mM EDTA, pH 8). Inclusion bodies were purified as described previously [23]. Briefly, cells were sonicated twice (each passage of three min cycles on ice; output 5, 50 % duty cycle) and then centrifuged for 15 min at 30,000 g at 4 °C. The pellet was resuspended in TE buffer (1 ml for 1 g), sonicated for 3 min on ice, and centrifuged for 15 min at 30,000 g at 4 °C.

The pelleted inclusion bodies were solubilized at 1 mg per 8 mL of TE-urea buffer (8 M urea, 50 mM Tris, 1 mM EDTA pH 8). After incubation for at least 4 hours at 4 °C with gentle agitation, the soluble inclusion bodies were centrifuged for 30 min at 30,000 g. Supernatants were then dialyzed overnight at 4 °C against water (Spectra/Por Dialysis Membrane MWCO 3,500). After 1 hour of ultracentrifugation at 100,000 g at 4 °C, urea, Tris and EDTA were added to the supernatants at the following final concentrations: 8 M urea, 25 mM Tris pH 8, and 1 mM EDTA. The solution was passed through a Vivaspin® 20 centrifugal concentrator (MWCO 30,000). Flow-throughs were dialyzed overnight at 4 °C against water (Spectra/Por Dialysis Membrane MWCO 3,500). After 40 min of ultracentrifugation at 100,000 g at 4 °C, the supernatant was frozen in liquid nitrogen and lyophilized. The powder was sollubilized in 500 µL of TE-urea buffer. Monomers and oligomers of Aß were separated by size-exclusion chromatography on a Superdex-75 10/300 GL Column. Column was equilibrated in phosphate-buffered saline (137 mM NaCl, 2.7 mM KCl, 10 mM Na_2_HPO_4_, 1.76 mM KH_2_PO_4_, pH 7.4) at 4°C. Column calibration was done by injecting 50 µl of Gel Filtration Standard (Biorad). The monomeric and oligomeric Aß fractions were pooled separately, and aliquots were quantified with a Bradford assay, frozen in liquid nitrogen, and conserved at -80 °C until use. 

### THT (Thioflavine T ) fluorescence assay

Aliquots of monomeric or oligomeric Aß were diluted to the appropriate final concentration (20 µM or 30 µM) in PBS. Each sample had a total volume of 100 µL and contained 20 µM thioflavin T dye. All kinetics measurements were carried out at 30 °C in 96-well plates. Data were recorded using a POLARstar Omega reader (BGM). Data points were recorded in cycles using the bottom-reading mode. A single cycle consisted of 10 s of orbital shaking (600 rpm) before fluorescence measurement, which was taken as the average of 10 flashes every 6 min. The dye was excited at 440 nm, and the fluorescence signal was recorded at 480 nm.

For cross-seeding assays, nucleation seeds were prepared from fibers obtained during the kinetics measurement described above. A 100 µL aliquot of each sample was sonicated for 5 min (output 5 and 50 % duty cycle). A 2 µL aliquot of sonicated fibers was added to 100 µL of 20 µM Aβ monomers or oligomers. 

### Bis-ANS fluorescence assays

The oG_37_CUR (50 μM) was fractionated by SEC on a Superdex-75 column in PBS. Absorbance at 215 nm and fluorescence at 480 nm in the presence of 1 µM bis-ANS were measured at t0. At different times during incubation in PBS with 5 mM DTT, the fluorescence in the presence of 1 µM bis-ANS was recorded. The dye was excited at 360 nm, and the fluorescence signal was recorded at 480 nm using an LS50B luminescence spectrometer (PERKIN ELMER).

### Circular dichroism

CD spectra were obtained in the far-UV region with a JASCO J810 spectropolarimeter. Samples were prepared as described above (see subsection Tht fluorescence assay) and then placed in a 0.1-cm pathlength quartz cuvette and monitored in continuous scan mode (260-190 nm). Data were averaged from 10 scans.

### IR spectroscopy

Fibrillation of Aß was generated over 15 days, in conditions described above (see subsection Tht fluorescence assay). 

Each sample (20 µl) was loaded on a germanium crystal (Specac, Orpington, UK) and dried with a stream of dry air. ATR-FTIR (Attenuated Total Reflectance Fourier-Transformed Infra-Red) spectra were recorded on a Nicolet Nexus 870 FTIR spectrometer equipped with a mercury cadmium telluride detector (Thermo Fisher Scientific, San Jose, CA, USA) with a spectral resolution of 4 cm−1 and a one-level zero filling. One hundred interferograms, representing an acquisition time of 3.5 min, were co-added. For clarity, the maximum absorbance for the amide I band has been normalized to the same value for all spectra shown.

### Transmission electron microscopy

A 5 μl aliquot of aggregates was adsorbed onto Formvar-coated, carbon-stabilized copper grids (200 mesh) for 10 min, washed 3 times with water, and dried with filter paper. Grids were then negatively stained with 10 µL of 2 % uranyl acetate, dried with filter paper and observed with a Philips TECNAI 12 Biotwin electron microscope at 80 kV.

### Cell culture and MTT assays

PC12 cells were cultured in RPMI 1640 medium (Sigma) supplemented with 10 % horse serum, 5 % fetal bovine serum, 0.1 % penicillin, and 0.1 % streptomycin on poly-D-lysine (PDL)-coated dishes. 

Aß monomers were diluted to 20 µM in PBS and incubated at 30 °C for 6 or 24 hours. G37C oligomers (oAß_G37C_UR) were diluted to 20 µM in PBS and incubated with 5 mM DTT at 30 °C for 6 or 24 hours. 

The viability of PC12 cells was measured using the Cell Proliferation Kit I (MTT) from Roche. PC12 cells (40,000 cells/well in 80 µL medium) were cultured on PDL-coated 96-well plates overnight. Aß samples (20 µL) aliquoted from the incubated Aß samples and diluted to the appropriate Aß concentration were added to the wells and incubated for 16 hours. The Aß concentration in the cell culture medium was kept at 0.5 µM. For control samples, the same volume of PBS was added to the wells. For the 3-(4,5-dimethylthiazol-2-yl)-2,5-diphenyltetrazolium bromide (MTT) reaction and measurement, the adsorption values at 550 nm were determined using a Tecan microplate reader. The viability of cells treated with PBS alone was set as 100%.

### Statistical Analyses

Data are expressed as means ± S.D. Statistical analyses were performed using one-way analysis of variance followed by unpaired Student’s *t* test.

## Results

### Single mutants of Aß_42_ dramatically alter Aß toxicity

**Figure 1:**
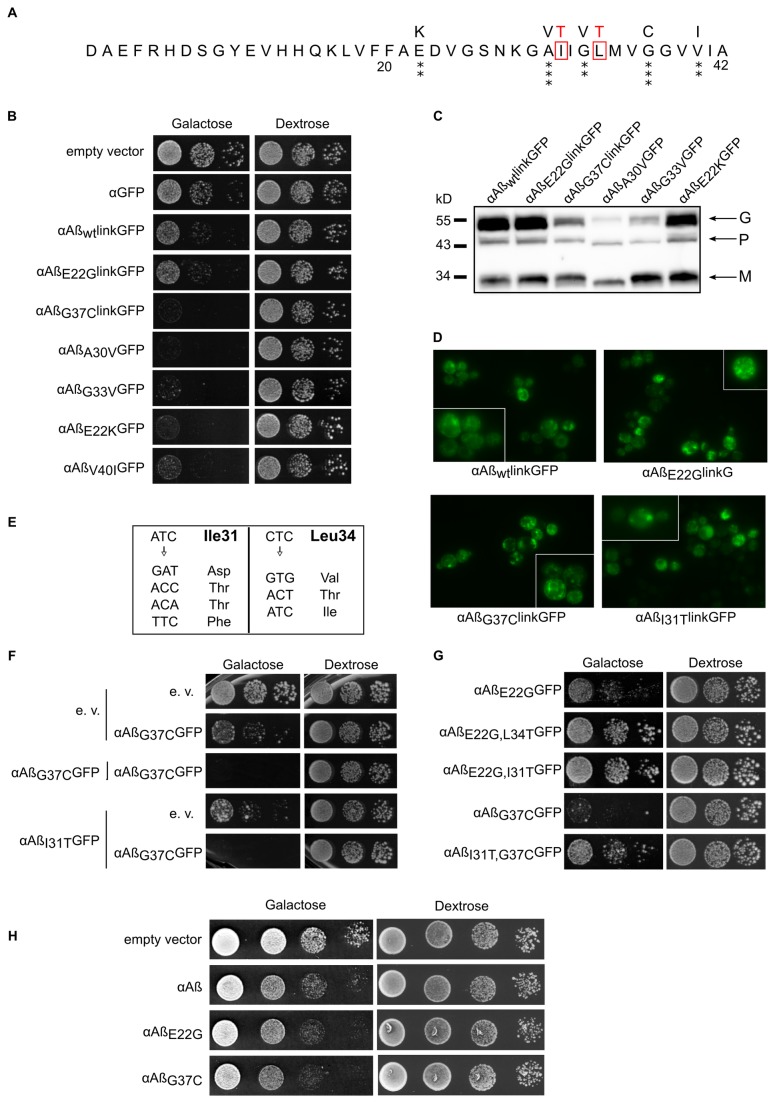
Cellular characterization of Aß_42_ mutants selected for their toxicity. **A**: Peptide sequences of Aß alleles. Amino acid substitutions in mutated alleles are shown at the top. The number of asterisks (*) is proportional to their toxicity in yeast. Non-toxic mutations are boxed in red. **B**: Comparison of the toxicities of Aß alleles in yeast. BY4742 cells carrying various plasmids were serially diluted and spotted on galactose (inducing) and dextrose (non-inducing) media. Cell growth was observed after 48h. **C**: Expression of mutated Aß. BY4742 WT cells expressing the highly toxic Aß alleles after 6 hours of induction were collected to extract total protein. Equal quantities of protein were separated by SDS-PAGE (12%), transferred to nitrocellulose membranes, and probed with an anti-Aß antibody (Santa-Cruz Biotechnology). The bands correspond to the precursor (P), glycosylated (G) and mature (M) forms. **D**: Aggregation patterns of GFP-tagged Aß alleles in WT yeast. WT cells were grown for 6 h in galactose medium to induce the expression of chimeric proteins and were examined by epifluorescence microscopy. **E**: Ile31 and Leu34 are essential for Aß toxicity. Site-directed mutagenesis of Ile31 and Leu34 was performed with degenerate primers. The resulting harmless mutants were sequenced. The nucleotide substitutions and the corresponding changes in amino acids are shown. **F**: Harmless mutations do not prevent toxicity in *trans*. BY4742 WT cells co-transformed by the indicated plasmids were serially diluted and spotted on inducing and non-inducing media. Growth was observed after 72 h. **G:** Harmless mutations prevent toxicity in *cis*. BY4742 WT cells transformed by the Aß double mutant were serially diluted and spotted on inducing and non-inducing media. Growth was observed after 48 h. H: Aß mutants are toxic without GFP. BY4742 deleted for a gene involved in Aß-GFP toxicity and carrying different alleles of Aß without GFP were serially diluted and spotted on galactose (inducing) and dextrose (non-inducing) media. Cell growth was observed after 48 h.

The mutagenesis of Aß yielded an average of 2.5 mutations per clone within the Aß coding sequence, which is consistent with our previous experiments [18]. Among the 6,000 clones analyzed, 54 were clearly affected in their capacity to form normal colonies when Aß was expressed, and the corresponding plasmids were sequenced. All the mutations are missense mutations (Figure S1). The most toxic single mutants in yeast are shown in Figure 1 (panel A). These single mutations were located within residues 18-42, which form a ß-strand (18-26)-turn-ß-strand (31-42) “U-shape” motif, as determined by solid state NMR [20]. The toxicity induced by these different alleles of Aß was estimated by a serial dilution assay (Figure 1, panel B). Interestingly, the selected mutants appeared to be much more toxic than the Arctic (E22G) mutation, which is itself slightly more toxic than the WT in our yeast model [17]. 

One mutant (E22K) was previously identified in an Italian familial case of Alzheimer’s disease [25]. *In vitro*, this mutation increases the rate of aggregation of synthetic Aß_42_ [26].These differences in toxicity (G37C > A30V> G33V> E22K> V40I> E22G> WT) could have been caused by different levels of expression of these Aß species within the yeast cells. This trivial explanation was definitively ruled out by a Western blot experiment (Figure 1, panel C). Interestingly, the ratios between the precursor, glycosylated, and mature forms remained roughly the same for the more toxic mutant G37C, indicating comparable secretion efficiencies, in G37C vs WT. Such a distribution would be expected to correspond to an equivalent distribution of the protein within different cellular compartments. This mutant (G37C) exhibited the same pattern of localization as the other non-toxic allele (I31T) tested (Figure 1 Panel D). It is therefore clear that the toxicity of these new Aß alleles was not due to a change in protein concentration or localization but rather to other parameters, such as its auto-assembly capacity.

In many cases, multiple mutations can be found within a mutagenized Aß sequence. Interestingly, among the 135 single changes obtained, we never observed any mutations of V12, L34, or I31 (data not shown). This result could have been due to a non-random distribution of the mutations. Alternatively, it might indicate that these aminoacids are crucial for Aß toxicity and that their mutation would be counter-selected in our screen (this screen was based on the gain of toxicity of Aß). We tested this hypothesis and created point mutations at these three positions. Site-directed mutagenesis was performed using degenerate oligonucleotides for the different amino acids (V12, L34 and I31). After mutagenesis of the V12 position, most of the clones obtained remained toxic, indicating that this aminoacid may be changed without a loss of toxicity (data not shown). In contrast, most of the L34 and I31 mutants were harmless. Sequence analysis of several clones revealed mutations in the first, second or third position within the codon (Figure 1, panel E). In all cases (data not shown), these mutations were non-synonymous (i.e., they led to a change in the primary structure of the peptide). Even conservative mutations, such as isoleucine for leucine, caused a significant change in Aß toxicity. 

Because our screen produced both harmful and harmless mutations, we were interested to determine whether these mutations could act both in *cis* (the two mutations being in the same peptide) and in *trans* (the two single mutants are produced separately in the same yeast cell). The co-expression of toxic mutants and WT Aß within the same yeast cell led to growth defects similar to those induced by the toxic mutant alone (data not shown). This dominant phenotype was also found when the harmless I31T and the harmful G37C mutant were co-expressed, demonstrating that non-toxic Aß mutants could not prevent Aß toxicity in *trans* (Figure 1, panel F). When both mutations were made in the same Aß protein, the double mutant was only slightly toxic, indicating that I31T and L34T prevent the formation of toxic species in *cis* (Figure 1, panel G). This finding is consistent with the hypothesis of more than one Aß folding pathway leading to different entities. 

As such the combination of GFP, particularly with the small Aß peptide may significantly bias the results of our screening. In our yeast expression system, we already know that expression of Aß without GFP lead to a modest toxic effect [17], making any toxicity measurement rather tricky. This is probably due to the role of GFP that stabilizes Aß and increase its cellular concentration [17]. However, when expressed in a euroscarf yeast strain selected for its increased sensitivity toward Aß-GFP (Vignaud et al., to be published), we could clearly observe and monitor growth impairment due to Aß expression. Importantly, Aß is not tagged in this experiment and we could confirm that Aß_G37C_ is more toxic than E22G itself slightly more toxic than the WT (Figure 1, panel h). These *in vivo* observations led us to further investigate the properties of these different Aß peptides *in vitro*. 

### Polymerization rate and fibrillar organization of Aß_42_ mutants

**Figure 2:**
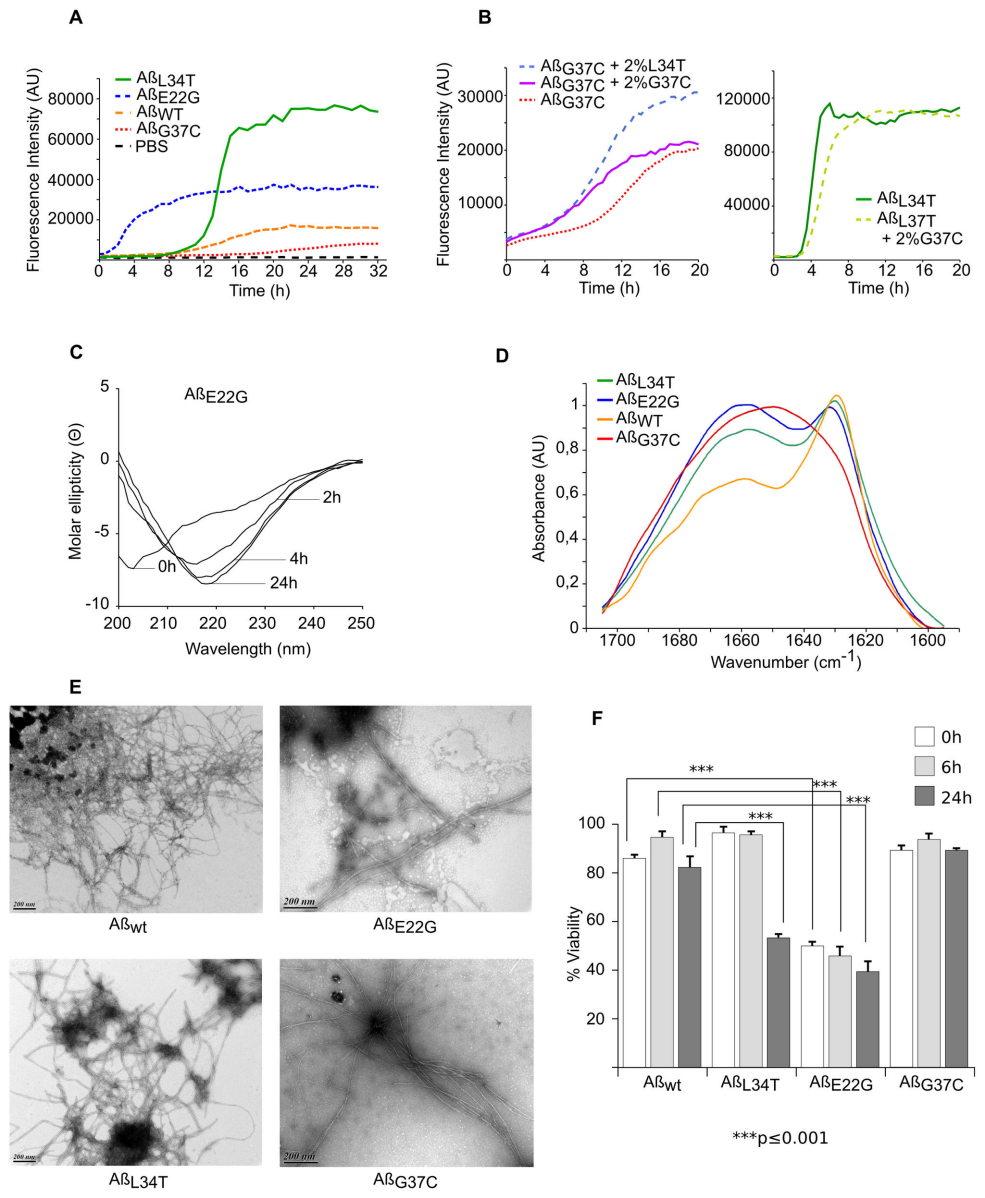
Auto-assembly of purified monomers. **A**: Fibrillation of Aß peptides monitored by ThT. Monomeric Aß_WT_ (orange), Aß_E22G_ (blue), Aß_G37C_ (red), or non-toxic Aß_L34T_ (green) peptides (20 µM) were incubated at 30 °C with 20 µM ThT in PBS. Background signal (dashed black), 20 µM ThT in PBS. **B**: Cross-seeding of monomeric Aß_G37C_ and Aß_L34T_. Left, 30 µM monomeric Aß_G37C_ alone (orange) or seeded with 2 % sonicated Aß_G37C_ (purple) or Aß_L34T_ (blue) fibers was incubated at 30 °C in 20 µM ThT. Right, 30 µM monomeric Aß_L34T_ with (light green) or without 2 % sonicated Aß_G37C_ fibers (dark green) was incubated under the same conditions. **C**: Structural changes in Aß_E22G_ monitored by CD spectroscopy. Far-UV spectra of Aß_E22G_ (20 µM) recorded at different times at 30 °C. **D**: ATR-FTIR spectra of Aß_42_. Spectra of Aß_wt_ (orange), Aß_E22G_ (blue), Aß_L34T_ (green) and Aß_G37C_ (red) (20 μM) were recorded after 15 days at 30 °C (all normalized to 1). **E**: Morphology of Aß fibers. During auto-assembly followed by ThT exposure, an aliquot of each peptide from the assembly plateau stage was negatively stained with 2 % uranyl acetate, loaded onto a coated grill, and analyzed by TEM. Scale bars: 200 nm. **F**: Toxicity of Aß variants in PC12 cells. At various times (0, 6 and 24 h) during fibrillation in PBS at 30 °C, 0.5 μM of each mutant Aβ was added to the medium. After 16 hours, cell viability was measured with MTT assays. The cell viability of cell treated with PBS was set as 100 %. The values represent the mean+S.D. of three experiments, plotted as solid bars (white, light gray, and dark gray for 0, 6, and 24 hours, respectively). ***p<0.001 compared with WT-Aß_42_

The different Aß peptides were expressed in *E. coli* and further purified, the final step being a size exclusion chromatography. The peak of Aß peptides eluted from the column as a monomeric species was flash-frozen. Different approaches may be used to follow Aß assembly, and we used ThT binding because it allowed us to follow fibrillation kinetics in real time. In this assay, all mutants (toxic E22G and G37C, harmless L34T and WT) exhibited the typical sigmoidal assembly curve (Figure 2, panel A). The results were consistent with a nucleation-dependent polymerization process. At the same concentration (20 µM), the lag time before the increase in fluorescence varied greatly among the mutants, from a few minutes (E22G) to several hours (G37C). This criterion was therefore not sufficient to predict the *in vivo* phenotype of each Aß mutant (i.e., the severity of the Aß toxicity in yeast cells). This is also the case for other Aß mutants. In particular, the D23N-Aß_40_ Iowa mutant and E22Q Dutch mutant exhibit enhanced rates of fibril assembly compared with wild-type Aß peptides [26]. Meanwhile, A21G Flemish Aß_40_ exhibits a much slower assembly rate, comparable with that of wild-type Aß [26]. 

At the same concentration and under the same experimental conditions (agitation, buffer, temperature, etc.), the different mutants plateaued at different levels. Similar differences in ThT fluorescence can be observed for one peptide when the experimental conditions vary [27] and may be explained by the proportion of the peptide that is able to auto-assemble and/or by structural variations in Aβ fibrils (indicating structural differences reminiscent of amyloid strain characteristics [28]). Such fibril characteristics can be passed on by seeding in a strain-like behavior [29]. The addition of fibril seeds (2 % sonicated L34T or G37C aggregates) to a solution of monomeric G37C shortened the lag time (Figure 2, panel B). Interestingly, the level of fluorescence depended on the type of fibrils added for the seeding and echoed the fluorescence reached by monomeric solutions incubated without seeding. Strikingly, monomeric L34T assembly was not stimulated (or was stimulated very poorly) by the presence of sonicated G37C fibrils (Figure 2, panel C), although it was stimulated normally by sonicated L34T aggregates (data not shown). This may indicate a low “seed” content in the G37C fibrils (leading to low ThT fluorescence) and/or a structural difference that created a “species barrier” [30]. 

We analyzed the structural changes at different times during the lag phase, the exponential increase, and the plateau, by circular dichroism (CD) spectroscopy. The CD spectra demonstrated a continuous decrease in the content of unordered (random coil) conformations and a concomitant increase in the content of β-structures (Figure 2, panel C). The time course of this switch was consistent with the aggregation of Aβ variants, as estimated using the thioflavin T fluorescence assay. The intensity of the negative peak at 218 nm depended on the mutant analyzed (data not shown) but was not correlated with the ThT maximum value or Aß toxicity in yeast. This lack of correlation may reflect a real property of amyloid fibrils formed by the different mutants. However, the measurement of the spectra might have been slightly distorted, as the observed changes in the spectra were the result of conformational changes and simultaneous aggregation with the formation of higher, partially insoluble, aggregates. 

The final aggregates were further characterized by ATR-FTIR and electronic microscopy. The analyses of the amide I region (1600-1700 cm^-1^) of the ATR-FTIR spectrum allowed us to identify the presence of parallel or anti-parallel ß-sheets, and random coil organizations. The aggregates formed by the WT, L34T, and E22G mutants showed typical parallel ß-sheet features, characterized by an absorbance maximum at 1630 cm^-1^ (Figure 2, panel D). A parallel β-sheet organization exhibits two bands, the most intense at around 1630 cm^-1^ and a less intense at 1651 cm^-1^, which was difficult to distinguish due to their overlap with random contributions [31,32]. This finding is consistent with the classical in-register parallel arrangement model based on NMR and site-directed spin labeling experiments [33,34]. The spectrum obtained with the G37C mutant was less conventional. The presence of a broad band indicates multiple contributions and can explain the lack of efficient cross-seeding with monomeric L34T mutant.

The ultrastructural organization of the aggregates was analyzed by electron microscopy of negatively stained samples. All the aggregates formed from the different Aß peptides exhibited a typical fibrillar structure. The morphology of these fibrils resembled each other closely, as the fibrils that formed appeared to be quite similar at this level of resolution (Figure 2, panel E). 

All these results converge toward a puzzling result: harmful (E22G, WT) and harmless (L34T) Aß_42_ behaved in the same way *in vitro*, and the most toxic mutant (G37C) formed also fibrils without clear identification of the parallel ß-sheets organization. This lack of correlation between toxicity and molecular organization of the monomers was further supported by the viability test in cell culture. We examined the relative toxicity of each Aß_42_ peptide using an MTT assay in PC12 cells (Figure 2, panel F). Prior to their addition to the cell culture, the monomeric Aß_42_ peptides were incubated for 0, 6, or 24 hours. The cells were then treated for 16 hours at a concentration of 0.5 μM Aβ in each cell well. At the end of this period, MTT reagent was added. The Arctic mutant was the most deleterious mutant, but, strikingly, the harmless L34T mutant appeared to be more toxic than the WT after 24 h, and the mutant that was the most toxic in yeast G37C was not more efficient than wt at reducing cellular viability in PC12 cells. Obviously, the results obtained *in vitro* with the purified monomeric fraction of the different Aß_42_ peptides are not consistent with the *in vivo* data.

### Toxic Aß mutants accumulate oligomers

**Figure 3:**
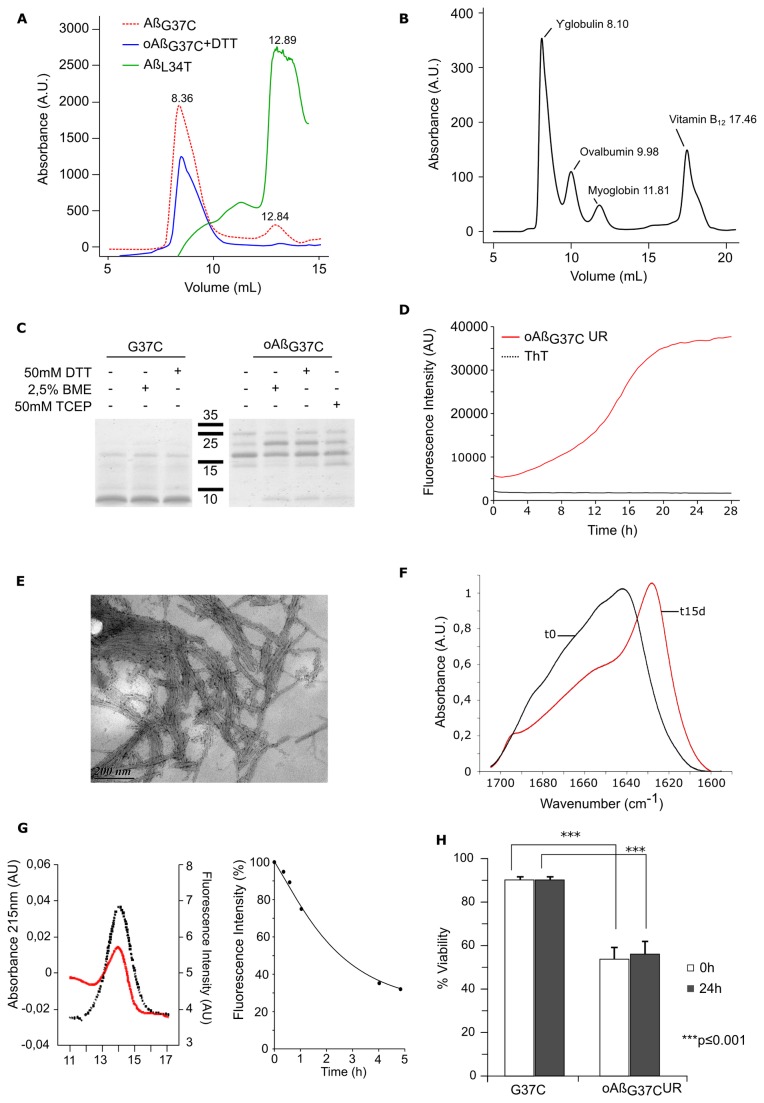
Isolation and characterization of G37C oligomers. **A**: Toxic Aß forms DTT resistant oligomers during purification. After dialysis, different species were eluted from a Superdex-75 column in PBS at 4 °C. Toxic Aß_G37C_ (red) and harmless Aß_L34T_ (green) were found in one or two peaks detected at 215 nm. The presence of DTT (5 mM) to the purified oligomeric fraction do not change its elution profile from a Superdex-75 column equilibrated in PBS + DTT (blue), **B**: Elution profile of Standards on the Superdex-75 column in PBS at 4 °C. **C**: Oligomeric urea-resistant Aß_G37C_ (oAß_G37C_UR) is not stabilized by S-S bonds. Equal quantities of oAß_G37C_UR (0.5 moles) were analyzed by Tricine-SDS-PAGE (15 %) under reducing (50 mM DTT or 50 mM TCEP or 2.5 % ß mercaptotethanol) or non-reducing (-) conditions. The same analysis was done for Aß_G37C_ with DTT and ß mercaptoethanol as reducing agent. **D**: Fibrillation of oAß_G37C_UR monitored by ThT fluorescence. 80 µM oAß_G37C_UR was incubated with 20 μM ThT at 30 °C. **E**: Morphology of oAß_G37C_UR fibers in TEM. After 7 days of fibrillation at 30 °C, oAß_G37C_UR was stained with 2 % uranyl acetate and observed by TEM. Scale bar: 200 nm. **F**: oAß_G37C_UR exhibits rich beta-parallel sheet structure in ATR-FTIR. ATR-FTIR spectrum of oAß_G37C_UR at t0 (black) and after 15 days at 30 °C (red). **G**: Conformational changes in oAß_G37C_UR during fibrillation in bis-ANS. 50 µM oAß_G37C_UR was loaded onto a Superdex-75 column in PBS. Left, the absorbance at 215 nm (red) and fluorescence in the presence of 1 µM bis-ANS (black) of oAß_G37C_UR loaded at t0. At different times during incubation at 30 °C in PBS + 5mM DTT, aliquots were taken for bis-ANS fluorescence analysis (right). **H**: Toxicity of oAß_G37C_UR in PC12 cells. The cytotoxicity of oligomeric oAß_G37C_UR in PC12 cells was determined with MTT assay. At time 0 (white) and after 24 hours of incubation in PBS (+ 5 mM DTT for oAβ_G37C_UR) (black), 0.5 μM of Aβ_G37C_ or oAβ_G37C_UR was added to PC12 cells. After 16 hours, cell viability was measured with MTT assays. The cell viability of cell treated with PBS was set as 100%. The values shown indicate the mean+S.D. of three experiments. ***p<0.001

Because none of these experiments helped us to understand the molecular basis of Aß toxicity, we decided to expand our analysis to the different species isolated during the last steps of our purification procedure. After ultrafiltration in the presence of urea, the flow-through was dialyzed against distilled water overnight. The corresponding protein was further lyophilized, dissolved in 8 M urea pH 8, and then filtered on a suitable SEC column to separate the Aß monomers from the urea. All species formed a monomeric peak. Interestingly, the non-toxic isoforms (L34T and I31T) each exhibited only one peak, corresponding to the monomeric form (unfolded, unable to bind ThT), whereas the other mutants produced peaks corresponding to higher molecular weight species (Figure 3, panel A). The ratio of the larger species peak to the monomeric peak was correlated with the toxicity of the isoform, with G37C exhibiting the highest ratio. Because this mutant was associated with the highest Aβ species content and was the most toxic, we focused our attention on this mutant. To rule out the possibility that these oligomers formed during the filtration, we repeated the separation in a buffer containing urea. When lyophilized protein resuspended in 8 M urea was loaded into a column equilibrated with PBS + 8 M urea and eluted with the same buffer, we again observed the same peak eluted in the same volume (data not shown). This indicated that the oligomers were generated during the dialysis and were not dissociated by 8 M urea. These oligomeric Aß_G37C_ urea-resistant species were named oAß_G37C_UR.

Cysteine may form disulfide bonds in proteins by oxidation, and these chemical links could stabilize a putative dimer or oligomer that could eventually incorporate the majority of G37C monomers. Such cross-linked intermediates would be resistant to urea and could explain the properties observed. We observed the same profile on Tricine-SDS-PAGE [35] in reducing (50 mM DTT, 50 mM TCEP or 2.5 % ß mercaptoethanol) or non-reducing conditions (Figure 3, panel B). The electrophoretic mobility was consistent with the formation of a trimer, but SDS-PAGE may induce the artifactual formation of high-molecular-weight Aß assemblies [36,37]. Monomeric Aß_G37C_ is mainly detected as a 4 kDa species but forms also higher species when loaded on SDS PAGE whatever reducing agent was added or not (Figure 3, panel B). However, because the SDS-PAGE profiles obtained with and without DTT were the same, the presence of a disulfide bond was very unlikely. To confirm this finding, we added DTT to the sample, equilibrated the gel filtration in PBS + DTT, and then loaded the oligomeric species onto the column. The presence of DTT did not change the elution profile, and the protein was found in the same fraction (Figure 3, panel C), indicating that the interactions leading to the formation of oligomers do not involve disulfide bonds. 

When incubated at 30°C under reducing conditions, these oAß_G37C_UR formed structures that interacted specifically with ThT (Figure 3, panel D). The kinetics of ThT binding were consistent with a model of nucleation-dependent polymerization, as is the case for monomeric peptides. This indicates that the oligomeric species purified at 4 °C (which do not bind ThT or bind poorly) can be converted into a different structure that could be either a different type of oligomer or a polymer resulting from its auto-assembly. Figure 3, panel E shows transmission electron microscopy (TEM) images of the structures formed during this kinetics assay. The species that bound ThT were clearly fibrillar. These fibrils assembled into large bundles of fibrils and seemed to interact laterally more efficiently than the fibrils made from monomeric proteins. 

In sharp contrast with other fibrils, fibrils made with oAß_G37C_UR always exhibited a strong capacity of assembly within the same plane. The capacities of these oligomeric species to bind ThT and to assemble into fibrillar structures resembled the auto-assembly mechanisms of the different monomers analyzed during this study. However, the FTIR spectra obtained from oligomeric oAß_G37C_UR at t0 and after auto-assembly were significantly different from those obtained from monomeric Aß, indicating that these entities adopted different structures. At t0, oligomeric species were mainly found as random coils. After the auto-assembly of the peptide, the amide I region in FTIR was characterized by the presence of the two characteristic peaks of anti-parallel ß-sheet structures, at 1630 and 1695 cm^-1^. This result indicates a switch from a random structure to an anti-parallel organization (Figure 3, panel F). This transitional shift from random coils to organized structures was further confirmed by measuring the fluorescence of ANS. Bis-ANS and ANS bind to exposed hydrophobic clusters on protein surfaces, resulting in a blue-shifted emission maximum and a significantly enhanced emission intensity [38,39]. This shift provides an interesting tool with which to follow the folding of a protein [39] and can thus be used to monitor the structural changes during Aß assembly. At t0, oAß_G37C_UR bound to ANS (Figure 3, panel G), as would be expected for an unstructured organization. When incubated at 30°C under conditions allowing the formation of ThT-binding species, this fluorescence decreased, indicating the burial of previously exposed hydrophobic domains (Figure 3, panel F).

The toxicity of these oligomers was then tested in PC12 mammalian cells. At t0, these oligomers appeared to be more toxic than the unincubated Aß_G37C_ (45 % vs 90 % viability) (Figure 3, panel H). The same results were observed after 24 hours of incubation (Figure 3, panel H).

## Discussion

The identification of the toxic species of Aß is the Holy Grail for many scientists in the field of Aß research. One of the difficulties these researchers face comes from the different tools used to track these elusive objects. Many different biochemical approaches have identified the existence of different amyloid intermediates, such as oligomers, proto-fibrils and annular aggregates [14,40]. These species are difficult to analyze because most of them are in equilibrium and in a metastable state that can be converted into a more stable organization. In addition to this intrinsic complexity, the most popular toxicity assay, based on the measurement of cell viability in the presence of Aß, adds a level of complexity due to the effects of the cell culture medium, which can change the properties of Aß species after several hours in culture. The best example of such interactions comes from the protocol used to isolate Aβ-derived diffusible ligands (ADDLs), which involves the incubation of Aß in F12 medium [41,42]. It is therefore difficult to make an unambiguous connection between *in vitro* and *in vivo* properties. Here, we used a reverse method based on a simple eukaryotic model to measure the toxicity of Aβ *in vivo*. These yeast cells, although not as complex as mammalian cells, are suitable for the study of amyloid properties such as prionization [43] and toxicity (for a review, see [44]). We predicted that the *in vitro* study of mutants selected for various levels of toxicity could reveal a type of biochemical signature that would explain the differences in their properties.

### Purified Aß_42_ monomers assemble into distinct fibrillar structures in vitro

**Figure 4:**
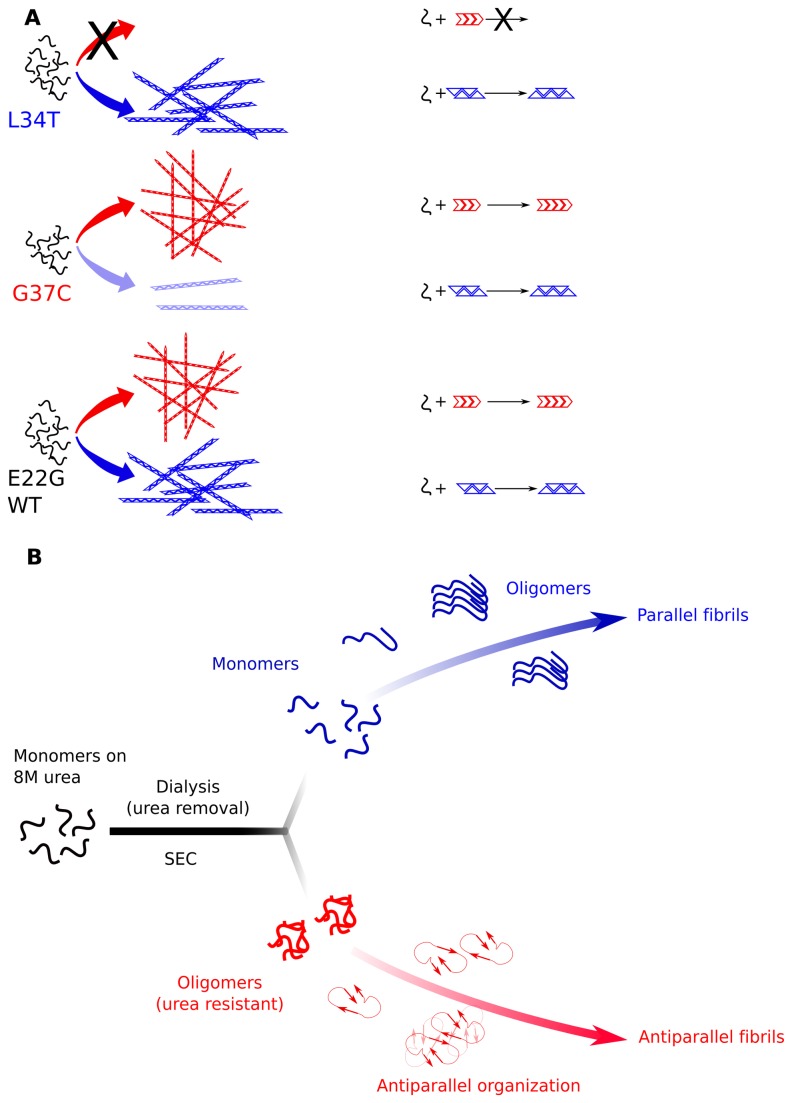
A working and hypothetical model of a dual fibrillar pathway. **A**: Purified monomers of non-toxic L34T form the only type of aggregate (represented as blue triangles) that can seed the fibrillation of all the peptides tested. The toxic G37C mutant preferentially forms a different fibrillar structure (red arrow) that can seed the aggregation of WT and the E22G mutant, but not L34T. This structure is stabilized by the G37C and E22G mutations and requires a particular folding environment, such as the slow removal of urea. The capacity to form these oligomers, which is the hallmark of toxic Aß mutants, leads to the further assembly into anti-parallel ß-sheet fibrils (**B**), which is a novel pathway.

We were able to isolate monomeric Aß via size-exclusion chromatography for all the different Aß peptides. These monomeric peptides, except that of the most toxic species (G37C), assembled into classical amyloid fibrils with high parallel ß-sheet contents. The G37C mutant also formed fibrillar structures that were able to bind the thioflavin, but the ATR-FTIR spectrum indicated the presence of different structural states, leading to a broad amide I band. Interestingly, G37C fibrils could not seed L34T assembly, whereas the opposite scenario (fibrils of L34T in the presence of monomeric G37C) seeded assembly efficiently. We hypothesized that L34T can form aggregates that can also incorporate G37C (represented by a blue triangle in Figure 4, panel A). In this model, the G37C mutant spontaneously assembles with a low efficiency into this structure and mainly forms a different type of aggregate (red arrow) that cannot be formed with the L34T mutant. Interestingly, WT Aß and E22G were efficiently cross-seeded with G37C and L34T fibrils (data not shown), indicating their ability to form both types of aggregate (Figure 4, panel A). 

Strikingly, monomeric G37C (isolated for its high toxicity in yeast cells) was less toxic than the other peptides when added to cultured cells. In the same assay, the harmless L34T mutant appeared to be much more toxic at 24 h than the WT-Aß_42_. Interestingly, this toxicity increased during fibril formation and the acquisition of the ß-sheet signature. These results contradict the *in vivo* analyses in yeast and may reflect a shortcoming of the cell assay, the yeast system used or both. Adding Aß to cell culture presents some bias [45] and does not reflect the complexity of the mechanisms in brain tissue. It might also be different from the toxicity of Aß formed within the cell. Obviously, yeast cells are not identical to neurons, but they can faithfully reproduce some of the events associated with intracellular toxicity, such as the interaction of Aβ with molecular chaperones. Regardless, there is no obvious link between the auto-assembly of the different monomeric species and cellular toxicity (monitored *in vitro* in cell culture by adding the peptides, or indicated by yeast viability during their intracellular production). This result suggests that most of the Aß toxicity relies on a different property. 

### Urea-resistant oligomers are a hallmark of toxic mutants

During our study, we discovered that Aß_42_ was able to form oligomeric species during the slow removal of urea by dialysis. The slow and gradual removal of the urea often allowed the protein to refold, mimicking the function of molecular chaperones that can assist Aß folding *in vivo*. All but the two harmless mutants were able to form oligomers. These oligomers could further assemble into a ß-sheet with anti-parallel organization. In a previous study, we also found that toxic mutants of the amyloid domain of Het-s display a characteristic anti-parallel structure in FTIR [19,32]. In the last 5 years, compelling evidences have shown that antiparallel amyloid structures were found in oligomers and to a lesser extend in certain fibrils. Remarkably, anti-paralleled organization was already evidenced for the IOWA (D23N) variant of Aß_40_, isolated as familial Alzheimer disease mutation [46]. The key point is that lot of these antiparallel structures were also correlated to toxicity [47]. The anti-parallel organization should favor fibril fragmentation, which in turn should result in the formation of smaller aggregates that would be more deleterious to cell viability, similar to the fibril fragments formed by mechanical treatment [48]. 

Aß_42_ oligomers are able to form anti-parallel β-sheet structures [49,50], and these oligomers are assumed to be intermediates in the pathway toward parallel ß-sheet fibril formation. However, no structural data can explain the formation of these species at the molecular level. The recent crystal structure of the Aβ_18-41_ fragment inserted into a single variable-chain immunoglobulin loop shows a tetrameric arrangement based on anti-parallel ß-sheets[51]. Interestingly, the two amino acids that are essential for Aß toxicity (L34 and I31) are also critical for this Aß packing. In particular, the four Leu34 side chains form a hydrophobic clamp, and a mutation of Leu34 would disrupt the central hydrophobic core of the tetramer and break the ß-sheets formed between adjacent dimers [51]. In this model, the two Ile31 side chains form a hydrophobic pocket that is essential for the formation of each dimer, and a mutation in Ile31 would thus lead to the inability to fold in this way. The essential roles of these two amino acids (Leu34 and Ile 31) in the anti-parallel packing of Aß are similar to their essential roles in forming toxic Aß. This correlation suggests that this folding may be somehow related to the formation of toxic species in a pathway that is clearly different from the auto-assembly pathway leading to parallel U-shape ß-sheet fibrils. This pathway evokes the structural organization of cylindrin, a toxic oligomer that can also pack into an out-of-register fibril that is toxic to mammalian cells [52].

These two pathways, if they exist in living cells, may contribute differently to Aß toxicity (Figure 4, panel B). Interestingly, the cytotoxicity of the urea-resistant G37C oligomer against culture cell was higher than monomeric unincubated G37C or WT. Thus it is plausible that such toxic oligomers, if produced in yeast, may partially explain the different toxic property of G37C mutant. The incapacity for L34T mutant to form such entities would also explain its lack of cellular toxicity.

In summary, our structure-toxicity study of Aß has identified new biological and structural aspects of Aβ folding. First, we demonstrated that toxic and non-toxic Aß_42_ monomers assemble into different types of fibrils. These fibrils are comparable to classical amyloid fibrils in terms of their fibrillar morphology, ThT binding ability, and ß-strand contents but differ in their capacity to seed the auto-assembly of the different Aß_42_ monomers.

Second, we identified a new type of oligomer that is formed during urea removal by dialysis. The presence of this oligomer is related to the toxic properties of Aß mutant peptides. The aminoacids crucial for the formation of these oligomers are also crucial for the formation of anti-parallel structures previously identified by other in a independent structural sudy.

Third, we have shown that these oligomers have the capacity to form fibrils that are rich in anti-parallel β-sheets and are different from the fibrils made *in vitro* from monomeric Aß. Our findings emphasize the role of a new, unidentified factor in amyloid toxicity and the need to revisit the pathologically relevant factors in Alzheimer’s disease. 

## Supporting Information

Figure S1 **After PCR mutagenesis and selection of harmfull mutants, 39 clones were sequenced.** The translation of mutated sequences is given here. Some mutants have more than one mutation.
(TIFF)
