# Supplementary figures and images for: A Structure-Toxicity Study of Aß42 Reveals a New Anti-Parallel Aggregation Pathway
Source: PLoS One. 2013 Nov 11;8(11):e80262. doi: 10.1371/journal.pone.0080262 (PMC3823702; doi:10.1371/journal.pone.0080262)

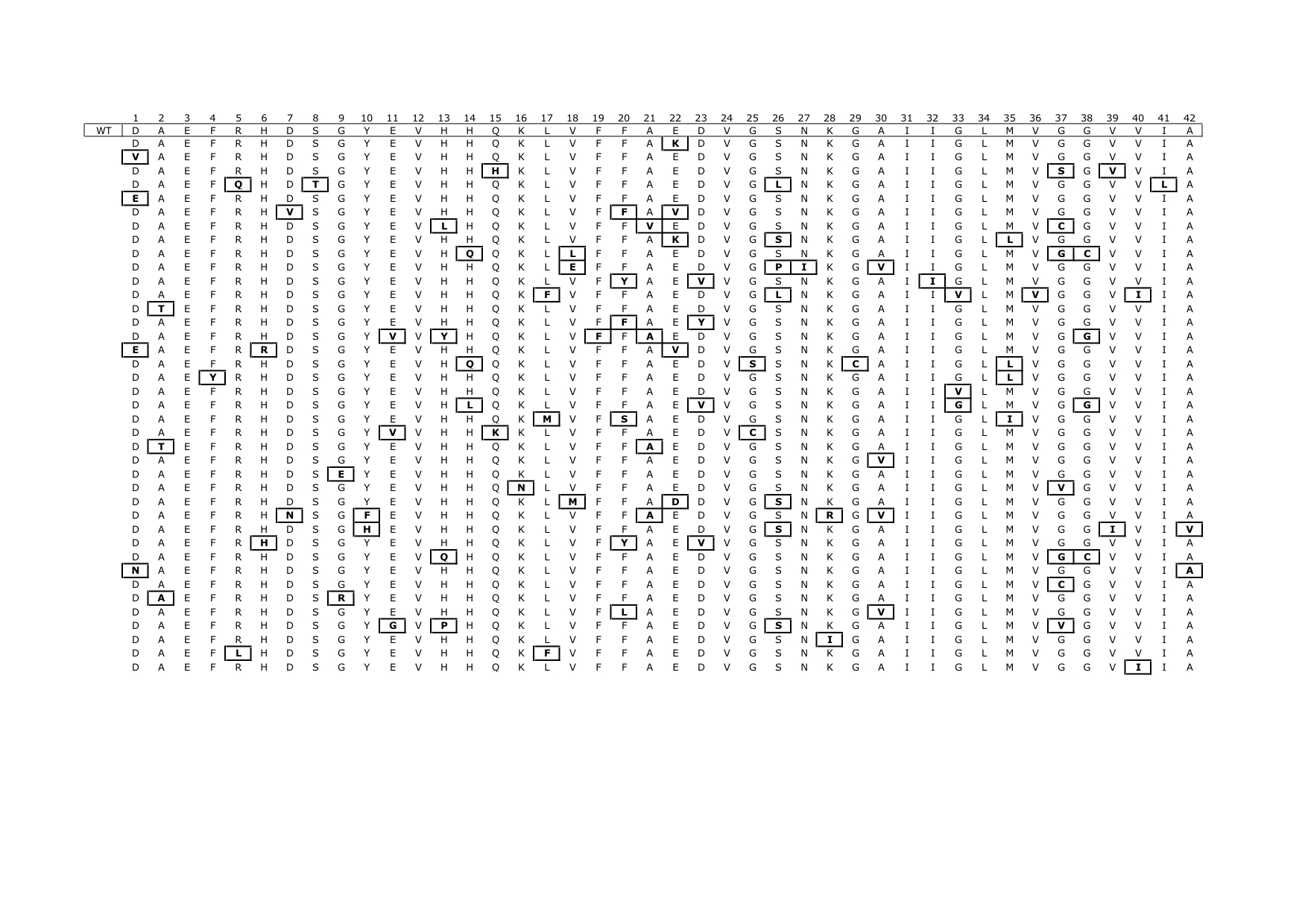

Supplement: Figure S1 — After PCR mutagenesis and selection of harmfull mutants, 39 clones were sequenced. The translation of mutated sequences is given here. Some mutants have more than one mutation. (TIFF) [file pone.0080262.s001.tiff]
